# Dynamic reorganization of photosynthetic supercomplexes during environmental acclimation of photosynthesis

**DOI:** 10.3389/fpls.2013.00513

**Published:** 2013-12-17

**Authors:** Jun Minagawa

**Affiliations:** Division of Environmental Photobiology, National Institute for Basic BiologyOkazaki, Japan

**Keywords:** acclimation, electron transfer, light-harvesting complex, non-photochemical quenching, phosphorylation

## Abstract

Plants and algae have acquired the ability to acclimate to ever-changing environments in order to survive. During photosynthesis, light energy is converted by several membrane protein supercomplexes into electrochemical energy, which is eventually used to assimilate CO_2_. The efficiency of photosynthesis is modulated by many environmental factors such as quality and quantity of light, temperature, drought, and CO_2_ concentration, among others. Accumulating evidence indicates that photosynthetic supercomplexes undergo supramolecular reorganization within a short time frame during acclimation to an environmental change. This reorganization includes state transitions that balance the excitation of photosystem I and II by shuttling peripheral antenna proteins between the two, thermal energy dissipation that occurs at energy-quenching sites within the light-harvesting antenna generated for negative feedback when excess light is absorbed, and cyclic electron flow that is facilitated between photosystem I and the cytochrome *bf* complex when cells demand more ATP and/or need to activate energy dissipation. This review will highlight the recent findings regarding these environmental acclimation events in model organisms with particular attention to the unicellular green alga *C. reinhardtii* and with reference to the vascular plant *A. thaliana*, which offers a glimpse into the dynamic behavior of photosynthetic machineries in nature.

## Introduction

Photosynthesis is the process of photochemical energy conversion that occurs via electron transport in the thylakoid membranes of chloroplasts, resulting in reduction of NADP^+^ in the stroma and concomitant generation of proton motive force across the thylakoid membranes. The NADPH generated with the electron flow and the ATP produced utilizing the proton motive force are required for assimilation of carbon dioxide in the Calvin-Benson cycle. Photosystem I (PSI) and photosystem II (PSII) represent charge separation devices to drive electron flow. Although these two photosystems originated from a common prototype, the contemporary PSI and PSII complexes are rather specialized and have major differences in organization of the light-harvesting system with regard to the reaction center, pigment composition and geometry, electron acceptors and donors, and several other features. I will begin this review by introducing the current knowledge of the components and structures of the two photosystems in their normal state, and then discuss their reorganization during various acclimation events in the later sections.

PSII and its light-harvesting complex proteins (LHCII) constitute a large chlorophyll (Chl)-protein supercomplex comprising more than 30 subunits. Light energy captured by LHCIIs is transferred to the central dimeric core complex, where it is trapped and utilized to drive electron flow from water to plastoquinone (PQ). In green plants, LHCIIs are formed by two layers, i.e., (1) major “more abundant” trimeric LHCII proteins, and (2) minor “less abundant” monomeric LHCII proteins (Dekker and Boekema, 2005). In the vascular plant *Arabidopsis thaliana*, there are three major trimeric LHCII proteins (type I–III) with 5, 4, and 1 isoforms (Lhcb1.1–1.5; Lhcb2.1–2.4; and Lhcb3.1), respectively (Jansson, 1999), whereas in the green alga *C. reinhardtii* there are four major LHCII proteins (type I–IV) with 5, 1, 2, and 1 isoforms (LhcbM3, −4, −6, −8, and −9; LhcbM5; LhcbM2 and −7; LhcbM1), respectively (Minagawa and Takahashi, 2004). The three minor monomeric LHCII polypeptides CP29, CP26, and CP24 are encoded by the *Lhcb4*, -*5*, and -*6* genes, respectively, in *A. thaliana* (Jansson, 1999), whereas *C. reinhardtii* contains only the first two (Teramoto et al., 2001).

Single-particle image analysis of electron micrographs revealed that these LHCII proteins are bound to both sides of the central dimeric core complex, with the core and the major LHCII trimers bordered by a few minor LHCII monomers (Dekker and Boekema, 2005). When spinach (*Spinacia oleracea*) thylakoid membranes are solubilized by n-dodecyl-β-D-maltoside (β-DM) (Boekema et al., 1995, 1998; Hankamer et al., 1997; Nield et al., 2000b), one LHCII trimer is bound strongly to each side of the core (C_2_S_2_ PSII-LHCII supercomplex), but when they are solubilized by n-dodecyl-α-D-maltoside (α-DM), the PSII-LHCII supercomplexes are organized as C_2_S_2_M_1−2_L_0−1_, or C_2_S_2_M_0_L_1−2_, wherein one to two moderately-bound LHCII trimers and/or one loosely-bound LHCII trimer, or one to two loosely-bound LHCII trimers, are associated with the C_2_S_2_-type supercomplex (Boekema et al., 1999). When *A. thaliana* thylakoid membranes are solubilized with α-DM and fractionated by gel filtration (Ruban et al., 2003) or on sucrose density gradients (Caffarri et al., 2009), the C_2_S_2_M_2_ organization is the largest type observed. These single-particle data from isolated detergent-solubilized PSII-LHCII supercomplexes were recently confirmed in observations of the organization directly within the thylakoid membranes by means of cryoelectron tomography (Daum et al., 2010; Kouřil et al., 2011).

**Figure 1:**
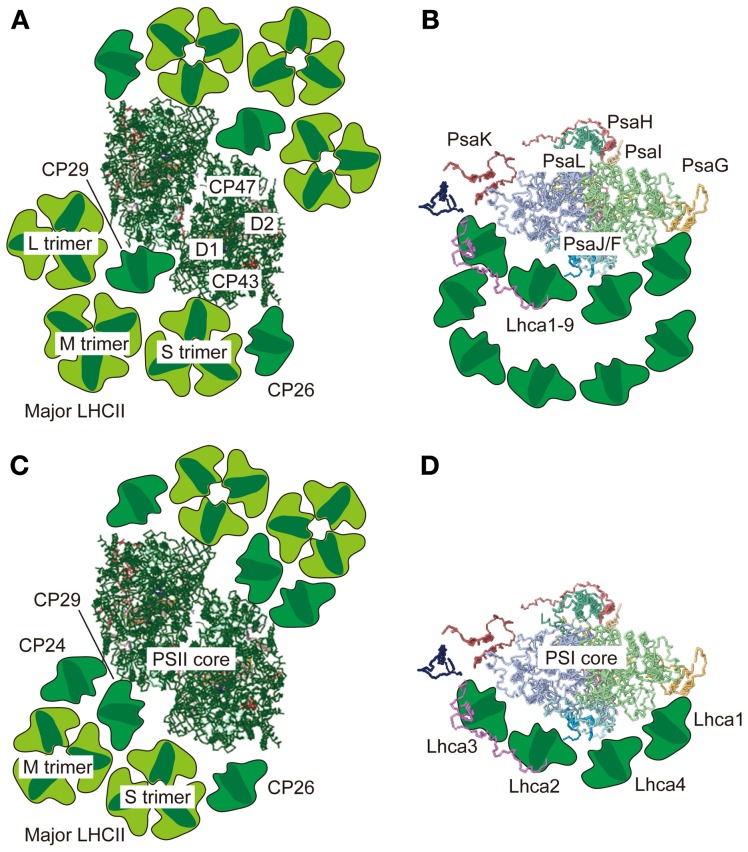
**Supramolecular organization of PSII-LHCII and PSI-LHCI supercomplexes in green algae and vascular plants.** Top views of the PSII-LHCII supercomplex **(A)** and the PSI-LHCI supercomplex **(B)** from *C. reinhardtii* based on single-particle image analysis by Tokutsu et al. (2012) and Drop et al. (2011), respectively. Top views of the PSII-LHCII supercomplex from spinach **(C)** and the PSI-LHCI supercomplex from pea **(D)** based on single-particle image analysis by Dekker and Boekema (2005) and crystallography of the PSI-LHCI supercomplex (Amunts et al., 2010), respectively. All top view images are from the lumenal side. The PSII and PSI core structures were taken from the coordinates determined by crystallography in 3ARC.pdb and 2WSC.pdb, respectively.

When the PSII-LHCII supercomplex from the green alga *C. reinhardtii* is prepared with a relatively high concentration of β-DM (50 mM; 2.6%), the C_2_S_2_ organization appears much as it does in vascular plants (Nield et al., 2000a). While a lack of M- and L-trimers in *C. reinhardtii* in an earlier report was tentatively ascribed to the absence of CP24 (Minagawa and Takahashi, 2004), which serves as a linker between PSII core subunits and an M-trimer in *A. thaliana* (Kovács et al., 2006; de Bianchi et al., 2008), both trimers were found in the more recent single particle analysis of the α-DM-solubilized PSII-LHCII supercomplex from *C. reinhardtii*, where three LHCII trimers were attached to each side of the core (the C_2_S_2_M_2_L_2_ PSII-LHCII supercomplex) (Tokutsu et al., 2012) (Figure 1A).

The supercomplex formed by PSI and its light-harvesting complex proteins (LHCI) is also a large Chl-protein complex comprising nearly 20 subunits. The PSI supercomplex collects light energy, converts it into electrochemical energy, and drives electron flow from plastocyanin (Pc) to ferredoxin (Fd). Whereas dimeric, trimeric, or tetrameric PSI cores have been reported in cyanobacteria (Boekema et al., 1987; Jordan et al., 2001; Watanabe et al., 2011), the eukaryotic PSI cores that harbor LHCIs are monomeric (Amunts et al., 2010). The association of LHCIs with a monomeric PSI core has been investigated using single-particle analysis, which revealed that in contrast to the case of the PSII supercomplex, LHCIs are asymmetrically bound to the PSI core in *C. reinhardtii* (Germano et al., 2002) and in spinach (Boekema et al., 2001) (Figure 1). The crescent-shaped “LHCI belt” was demonstrated to be associated with the side of the PsaF/J subunits in a 3.3 Å crystal structure of the PSI-LHCI supercomplex from pea (*Pisum sativum*) (Amunts et al., 2010). The other side of the core is unoccupied under normal conditions, exposing the PsaH/I/L subunits (Figure 1D), but could dock the mobile LHCII(s) under “State 2” conditions as described below. In vascular plants, the “LHCI belt” is formed by the four LHCI proteins in the order of Lhca1, −4, −2, and −3 (Figure 1). In *C. reinhardtii*, however, the “LHCI belt” is double layered and 9 LHCI proteins in total, encoded by the *Lhca1–9* genes (Minagawa, 2009), are attached to the side of the PsaJ/F subunits (Drop et al., 2011) (Figure 1B).

Because plants and algae typically do not have the means to escape adverse environmental conditions such as hot/cold temperatures, drought, high light (HL)/low light (LL), high/low CO_2_ concentration, etc., the ability to acclimate is essential if they are to survive in their niche. Acclimation of the photosynthetic machinery is especially important for photosynthetic organisms to optimize their photosynthetic performance and to protect their photosynthetic machinery from photooxidative damage in the natural environment, where the quality and quantity of light fluctuates over time. This review presents an overview of the emerging evidence that photosynthesis is acclimated to environmental conditions via dynamic reorganization of photosystem supercomplexes and super-supercomplexes. This reorganization is observed during state transitions, alternating between light-harvesting and energy dissipating modes, and switching between types of electron flow. I focus on studies in model organisms (Gutman and Niyogi, 2004), such as the unicellular green alga *C. reinhardtii* and the vascular plant *A. thaliana*, and refer readers to other reviews for more comprehensive information about the acclimation events themselves including state transitions (Lemeille and Rochaix, 2010; Minagawa, 2011), excess energy dissipation (Horton et al., 2008; Li et al., 2009; de Bianchi et al., 2010), and photosynthetic electron flow (Kramer et al., 2004; Finazzi, 2005; Shikanai, 2007; Alric, 2010; Johnson, 2011).

## State transitions

Each of the two charge-separation devices—PSI and PSII—in the thylakoid membranes has a distinct pigment system with unique absorption characteristics. Thus, an imbalance of energy distribution between the two photosystems tends to occur in natural environments, where light quality and quantity fluctuate with time (Allen, 1992; Bellafiore et al., 2005). Since the two photosystems are connected in series under normal conditions, plants and algae constantly need to balance their excitation levels to ensure optimal efficiency of electron flow. State transitions take place under such conditions to balance the light-harvesting capacities of the two photosystems in order to minimize unequal distribution of light energy. State 1 occurs when PSI is over-excited and the mobile antennas are more associated with PSII to correct the imbalance, which can be monitored based on a higher Chl fluorescence yield at room temperature. Conversely, State 2 describes the arrangement when PSII is over-excited and the mobile antennas are accordingly more associated with PSI; State 2 is characterized by a lower Chl fluorescence yield at room temperature.

**Figure 2:**
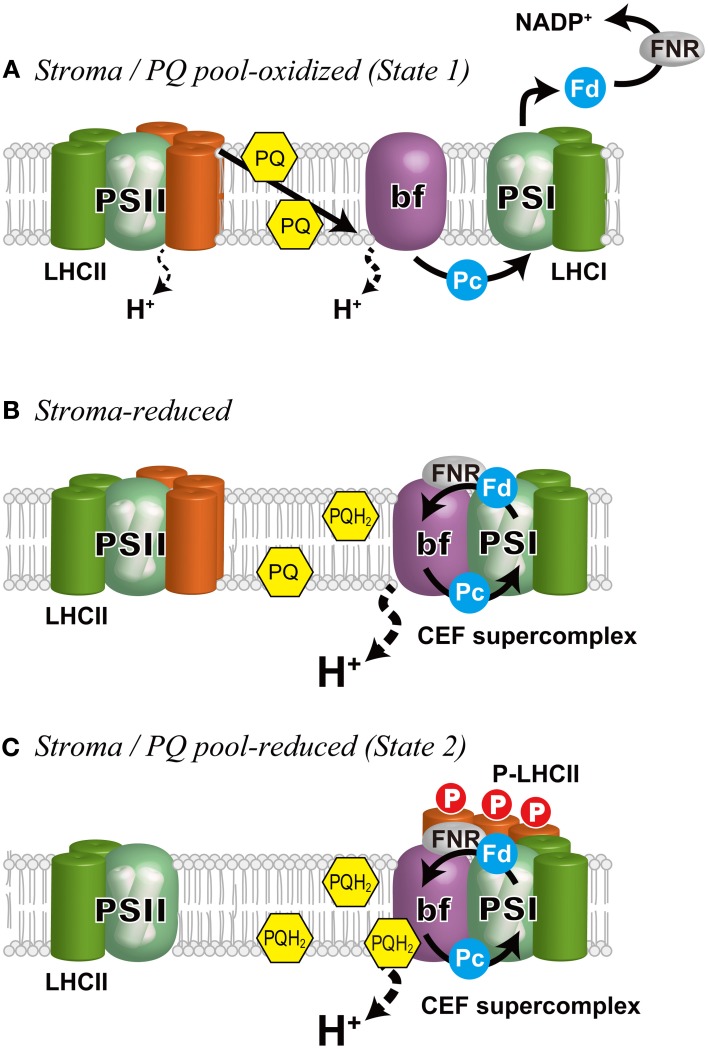
**Schematic representation of the regulation of state transitions and electron flow in *C. reinhardtii*. (A)** *Stroma/PQ pool-oxidized*, when PSI is preferentially excited and the stroma and the PQ pool are oxidized. Under these conditions, LHCIIs are bound to PSII (State 1). The photosynthetic electron flow proceeds in LEF mode generating NADPH as well as a proton gradient across the thylakoid membrane that is used for ATP production. **(B)** *Stroma-reduced*, when the stroma is reduced, CEF is induced (Takahashi et al., 2013). The cytochrome *b*_6_*f* complex and ferredoxin NADP^+^ reductase (FNR) are associated with PSI to form a super-supercomplex (CEF supercomplex). **(C)** *Stroma/PQ pool-reduced*, when the PQ pool is further reduced by the elevated CEF, migration of the mobile LHCIIs (*orange*) occurs on the PsaH side of PSI that is opposite the LHCI belt to establish State 2. bf, the cytochrome *b*_6_*f* complex; Fd, ferredoxin; Pc, plastocyanin.

Although the core concept (Bonaventura and Myers, 1969; Murata, 1969) and the molecular mechanisms of regulation, including the involvement of Cyt *bf* (Wollman and Lemaire, 1988), binding of PQH_2_ to the Qo-site of Cyt *bf* (Vener et al., 1997; Zito et al., 1999), and redox-dependent LHCII kinase (Depège et al., 2003) (Figure 2) have been established, investigation into the supramolecular reorganization of PSII and PSI has begun only recently, thanks to advancements in genetic and biochemical studies in two model organisms: the green alga *C. reinhardtii* and the vascular plant *A. thaliana*. In particular, many of the recent findings have been from *C. reinhardtii*. This is due, in part, to the fact that as much as 80% of the LHCII is mobile during state transitions in this green alga (Delosme et al., 1996), whereas only 20–25% of LHCII migrates in vascular plants (Allen, 1992).

### Reorganization of PSII supercomplex during state transitions

Traditionally, the PSII supercomplex has been thought to be reorganized during a State 1-to-2 transition such that some LHCIIs that form the peripheral antenna of PSII are detached upon their phosphorylation. The molecular details of this reorganization have been studied in *C. reinhardtii* (Iwai et al., 2008). Three PSII fractions corresponding to a PSII core complex, a PSII-LHCII supercomplex, and a multimer of the PSII-LHCII supercomplex were affinity-purified from a mutant expressing His-tagged CP47. Gel filtration and electron microscopy showed that the PSII-LHCII supercomplex is predominant in State 1, whereas the core complex is predominant in State 2, indicating LHCIIs are dissociated from PSII upon a State 1-to-2 transition. Moreover, in State 2, while most of the free LHCIIs are phosphorylated, most of those bound to the PSII-LHCII supercomplex are unphosphorylated, except for LHCII type I, which was found to be strongly phosphorylated in the supercomplex. The PSII subunits including the CP43 and D2 proteins in the core complex are mostly phosphorylated. Based on these findings, Iwai et al. (2008) hypothesized that (1) unphosphorylated LHCIIs stabilize the PSII complex (State 1); (2) the phosphorylation of LHCII type I in the major LHCII trimer triggers the monomerization of the supercomplexes; and (3) the phosphorylation of CP26 and CP29, as well as the PSII core subunits D2 and CP43, induces the detachment of the LHCIIs from PSII (Iwai et al., 2008). Although neither CP26 nor CP29 is phosphorylated while associated with the PSII-LHCII supercomplex, they are both phosphorylated when dissociated from PSII, suggesting that their dissociation is possibly caused by their phosphorylation. The minor monomeric LHCIIs border the major LHCII trimers and the PSII core (Harrer et al., 1998; Yakushevska et al., 2003) as shown in Figure 1, and the hyperphosphorylated residues in CP29 are indeed found at the interface of the PSII core and the peripheral antenna proteins (Turkina et al., 2006). Therefore, the phosphorylation of CP26 and CP29 likely triggers undocking of the entire peripheral antenna during the State 2 transition. It should be noted that the minor monomeric LHCIIs are also shuttled to PSI, acting as a linker between PSI and major trimeric LHCII during a transition to State 2 in *C. reinhardtii* (Takahashi et al., 2006; Tokutsu et al., 2009), although this is not the case in vascular plants (Galka et al., 2012).

Recently, the remodeling of PSII supercomplex during state transitions was also studied in *A. thaliana* (Dietzel et al., 2011). Although the dissociation of LHCIIs from PSII-LHCII supercomplex correlates with the time range of the transition from State 1 to State 2, the remodeling process of the PSII-LHCII megacomplex was unaffected in the *A. thaliana stn7* mutant, which is deficient in the kinase responsible for LHCII phosphorylation (Bellafiore et al., 2005). The authors therefore concluded that PSII-LHCII megacomplex remodeling precedes the migration of LHCII from PSII to PSI, but is independent of LHCII phosphorylation and dependent on phosphorylation of a PSII core subunit CP43, which, as they found, is not dependent on the STN8 kinase previously reported to phosphorylate the PSII core subunits (Bonardi et al., 2005). Although the results from *C. reinhardtii* and *A. thaliana* show differences at some points, prompting the two research groups to propose similar but different models for state transitions, and these differences could potentially reflect differences between green algae and vascular plants, both studies indicate that phosphorylation of a PSII subunit CP43 is prerequisite for remodeling of PSII-LHCII supercomplex that precedes migration of LHCII from PSII to PSI during a state transition.

### Reorganization of PSI supercomplex during state transitions

Scheller and his colleagues studied the association of the mobile LHCII with PSI via cross-linking and antisense approaches. *A. thaliana* plants without PsaH and PsaL (Lunde et al., 2000), as well as those without PsaO (Jensen et al., 2004), were highly deficient in state transitions. Since these small PSI subunits are located on a vacant side of the PSI core, opposite from the LHCI belt (Amunts et al., 2010) (Figure 1), these PSI subunits were hypothesized to constitute a specific binding site for the mobile LHCII. The association of LHCII with PSI was first biochemically demonstrated in the *A. thaliana psae1-1* mutant: a fraction of LHCII was associated with PSI when the mutant plants were exposed to LL conditions (State 2-favoring), giving rise to a high-molecular-mass protein-pigment complex (Pesaresi et al., 2002). This large complex, however, seemed to be an aggregated product, because the mutant did not show state transitions, probably due to its low level of PsaH. The next attempt to observe LHCII with PSI was via crosslinking of the mobile LHCII proteins with the PSI-LHCI supercomplex in *A. thaliana* (Zhang and Scheller, 2004). More of the major LHCII proteins, including Lhcb1 and -2, were crosslinked to the PsaH, PsaI, and PsaL subunits in State 2 than in State 1. Further information was provided in a study on *C. reinhardtii*, wherein the PSI-LHCI-LHCII supercomplex isolated from State 2 cells contained two minor monomeric LHCII proteins, CP26 and CP29, and one major trimeric LHCII protein, LhcbM5, suggesting a pivotal role for the minor monomeric LHCII in state transitions in green algae (Takahashi et al., 2006).

The significance of the minor LHCII in state transitions in *C. reinhardtii* was supported by an RNA interference (RNAi) study in which the levels of the two minor LHCII proteins, CP29 and CP26, were individually reduced (Tokutsu et al., 2009). Both the CP29 and CP26 RNAi mutants underwent reductions in the PSII antenna size during a State 1-to-2 transition, as reflected by non-photochemical quenching (NPQ) of fluorescence, low temperature fluorescence spectra, and functional absorption cross section data. However, the LHCIIs undocked from PSII did not re-associate with PSI in the CP29-RNAi mutant, as evidenced by the fact that the antenna size of PSI was not complementarily increased. By contrast, the mobile LHCIIs in the CP26-RNAi mutant did re-associate with PSI, such that a PSI-LHCI-LHCII supercomplex could be visualized on a sucrose density gradient (Tokutsu et al., 2009). These results thus clarify that CP29, and not CP26, is crucial when mobile LHCIIs re-associate with PSI under State 2 conditions in *C. reinhardtii*.

In *A. thaliana*, the identities of LHCII trimers and specific LHCII polypeptides involved in state transitions have been controversial. At one time it was thought that the M-trimer, where Lhcb3 is almost exclusively found (Hankamer et al., 1997), was not involved in state transitions because Lhcb3 is absent from stroma lamellae under State 2 conditions (Bassi et al., 1988). However, a later report indicated that although PSII performance is not altered in a knockout *A. thaliana* mutant lacking Lhcb3, in which Lhcb1 and/or Lhcb2 replace Lhcb3 in the M trimer, the rate of transition from State 1 to State 2 is increased, suggesting that the main function of Lhcb3, and thus the M-trimer, is to modulate the rate of state transitions (Damkjaer et al., 2009). Recently, Galka et al. (2012) succeeded in purifying from *A. thaliana* and maize the most complete PSI-LHCI-LHCII supercomplex reported so far, finding that it contained a LHCII trimer with Lhcb1 and 2, but not Lhcb3. Based on the relative accumulation of the Lhcb1-2 isoforms in the PSI-LHCI-LHCII trimer complex, they concluded the LHCII is neither S- nor M-trimer and tentatively speculated that it could be an L-trimer, which is loosely bound to PSII under State 1 conditions. The fluorescence analyses indicated that excitation energy migration from mobile LHCII to PSI was rapid and efficient, and the quantum yield of photochemistry in the PSI-LHCI-LHCII supercomplex was unaffected with respect to PSI. These facts let them to suggest that rather than thinking of the mobile pool of LHCII as a part of PSII that detaches under State 2 conditions, it would be more accurate to consider it to be PSI's own antenna system that can migrate to PSII under State 1 conditions. The 2D structure of such a PSI-LHCI-LHCII supercomplex in *A. thaliana* was visualized by single particle analysis of electron micrographs (Galka et al., 2012), where a LHCII trimer was found to be bound near the PsaH/I/L site. In contrast to this observation in a vascular plant, Barber and colleagues located earlier a smaller density near PsaH, which they assigned to CP29 in *C. reinhardtii* (Kargul et al., 2005).

## Thermal dissipation of excess absorbed energy

In nature, unexpected changes in light intensity can lead to overexcitation of the photosystems, resulting in the accumulation of harmful reactive oxygen species (Li et al., 2009). Plants and algae have developed protective NPQ mechanisms that alleviate such photooxidative stress. Among these mechanisms, qE quenching—downregulation of the light-harvesting capacity of PSII—thermally dissipates excess light energy captured by PSII as a negative feed-back mechanism for the elevated electron flow. Thanks to the genetic evidence provided first by *C. reinhardtii* and then by *A. thaliana* mutants with modified NPQ capacity (Niyogi et al., 1997a, 1998), great progress has been made in elucidating the site and the mechanism of qE [see (Horton et al., 2008; Li et al., 2009; de Bianchi et al., 2010) for reviews]. In vascular plants, qE is induced upon lumenal acidification, which activates the xanthophyll cycle and a qE effector PsbS (Horton et al., 1996; Müller et al., 2001). The xanthophyll cycle, deepoxidation of violaxanthin to antheraxanthin, and then further to zeaxanthin, is catalyzed by violaxanthin deepoxidase, which is activated by low pH (Demmig-Adams and Adams, 1992). For the activation of PsbS, low pH is sensed by two glutamic acid residues on PsbS (Li et al., 2000, 2004). The elevated zeaxanthin content (Johnson et al., 2011) and protonation of PsbS (Kereiche et al., 2010) decrease the formation of ordered semi-crystalline arrays of PSII supercomplexes, leading to an increase in the fluidity of the thylakoid membranes (Goral et al., 2012). This membrane “phase transition” facilitates dissociation of several LHCII proteins forming the outer layer of the PSII-LHCII supercomplex (Betterle et al., 2009) and aggregation of the dissociated LHCII (Kiss et al., 2008; Johnson et al., 2011), which probably allows for the conformational change within the major LHCII (Ruban et al., 2007) and/or the minor LHCII (Ahn et al., 2008) to generate energy-quenching site(s).

Although both zeaxanthin and PsbS are thought to have crucial roles in qE quenching in vascular plants, the green alga *C. reinhardtii* does not express the PsbS protein (Finazzi et al., 2006; Bonente et al., 2008), even though the *PsbS* gene is present (Anwaruzzaman et al., 2004), and a mutant deficient in violaxanthin de-epoxidase activity still shows qE quenching (Niyogi et al., 1997a,b). While PsbS is present even in LL-grown plants (Demmig-Adams et al., 2006), and therefore provides constitutive photoprotection, qE in *C. reinhardtii* is not activated immediately upon exposure to HL. The activation of qE in *C. reinhardtii* requires prolonged exposure to HL (Niyogi et al., 1997a) or low CO_2_ conditions (Förster et al., 2001), suggesting that algae have a distinct mechanism for qE induction and activation.

Niyogi and colleagues reported that the *C. reinhardtii npq4* mutant, which is deficient in an ancient LHC protein LHCSR3, induces little qE quenching (Peers et al., 2009). The genes for LHCSR3 (*Lhcsr3.1* and *Lhcsr3.2*), formerly known as LI818 (Gagne and Guertin, 1992), encode a 25-26 –kDa integral membrane protein whose expression is induced under HL (Richard et al., 2000), low CO_2_ (Miura et al., 2004), or low iron (Naumann et al., 2007) conditions. While PsbS cannot bind pigments, LHCSR3 is capable of binding Chl *a* and *b*, as well as xanthophylls (Bonente et al., 2011) Furthermore, a recombinant LHCSR3 polypeptide reconstituted with Chl and xanthophylls is capable of dissipating excitation energy in a low pH buffer, suggesting that this protein is the primary quenching effector in *C. reinhardtii* (Bonente et al., 2011). In addition to this possible “specialized” antenna protein involved in quenching modulation, genetic analysis in *C. reinhardtii* has led to the identification of other LHCs responsible for quenching. Depletion of one of the major trimeric LHCII proteins LHCBM1 in the *npq5* mutant (Elrad et al., 2002) decreases its capacity for thermal energy dissipation. It is possible that other gene products in addition to LHCSR3 and LHCBM1 may also be involved in HL acclimation in *C. reinhardtii*.

**Figure 3:**
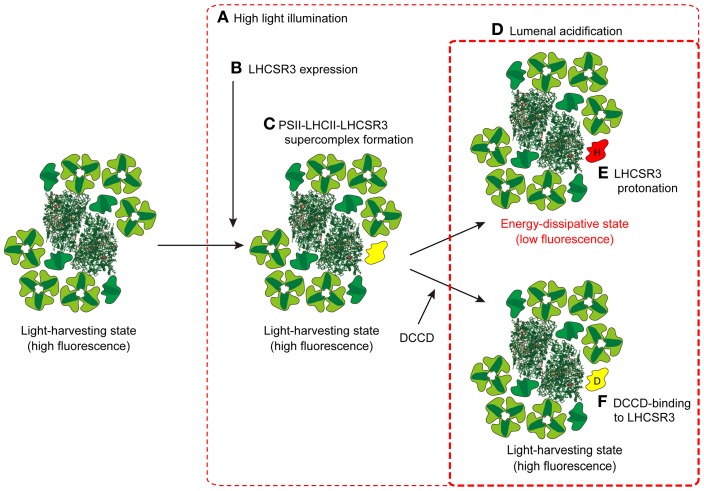
**A model for the induction of qE in *C. reinhardtii*.** When *C. reinhardtii* cells are exposed to HL for several hours **(A)**, the expression of LHCSR3 is induced **(B)**. LHCSR3 (*yellow*) is then associated with the PSII-LHCII supercomplex to form the PSII-LHCII-LHCSR3 supercomplex **(C)**. Though the PSII-LHCII-LHCSR3 supercomplex is still in a light-harvesting state under dark or LL conditions, it becomes energy-dissipative upon protonation of LHCSR3 (*red*; “H” denotes protonation) **(E)** in the acidified thylakoid lumen under HL conditions **(D)**. DCCD-binding to LHCSR3 (*yellow*; “D” denotes DCCD) inhibits the conversion of PSII-LHCII-LHCSR3 supercomplex from the light-harvesting state to the energy-dissipative state **(F)**. The crystal coordinates were obtained from the Protein Data Bank: PSII core, 3ARC; LHCII, 2NHW; and PSI-LHCI supercomplex, 2WSC.

A recent study examined where LHCSR3 is localized in the thylakoid membranes, and whether it dissipates energy captured by PSII (Tokutsu and Minagawa, 2013). By comparing the PSII-LHCII supercomplex from wild type (WT) *C. reinhardtii* cells and those of *npq4*, LHCSR3 was found to be present only in the PSII supercomplex from HL-grown WT cultures, and not in that from LL-grown WT or HL-grown *npq4*. The purified PSII-LHCII-LHCSR3 supercomplex was in a high-fluorescence state at a neutral pH (7.5), as evaluated by single-photon counting, but in an energy-dissipative state at pH 5.5, similar to the effect of lumenal acidification following HL illumination of thylakoid membranes. The switching from a light-harvesting state to an energy-dissipative state observed in the PSII-LHCII-LHCSR3 supercomplex was sensitive to dicyclohexylcarbodiimide (DCCD), a protein-modifying agent specific to protonatable amino acid residues. It is therefore likely that the association of LHCSR3 with the PSII-LHCII supercomplex is a reorganization necessary to dissipate excess absorbed energy under HL conditions in *C. reinhardtii* (Figure 3).

## Cyclic electron flow around photosystem I

**Figure 4:**
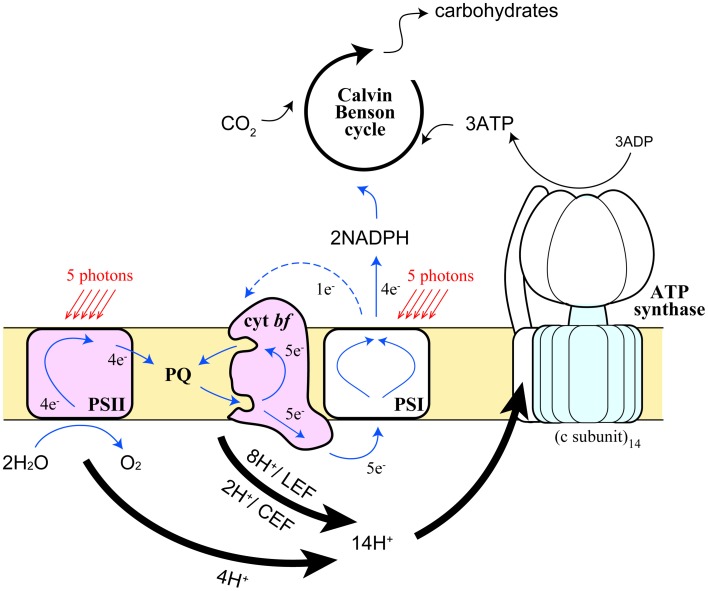
**Schematic representation of the movement of protons and electrons by the photosynthetic electron transport chain in the chloroplast.** Pathways and stoichiometry of light-driven electron transport, proton translocation, and ATP synthesis on the thylakoid membranes are shown. *Thick arrows* represent the pathways of protons, *blue solid* and *blue dashed arrows* represent LEF and CEF, respectively.

Electrons generated in the photosystems flow into two different pathways in the thylakoid membranes—linear electron flow (LEF) from water to NADP^+^ via PSII and PSI in series, and CEF around PSI (Arnon et al., 1958) (Figure 4). Although it is crucial to achieve the proper balance (3:2) of ATP and NADPH in the stroma in order to assimilate CO_2_ in the Calvin-Benson cycle, this balance cannot be achieved by LEF alone (Allen, 2003). As illustrated in Figure 4, 4 protons are released to the lumen and 2 molecules of reduced PQ (PQH_2_) are released to the intersystem pool from PSII, where 4 turnovers of PSII occur upon capturing 5 photons, assuming the quantum yield of the PSII photochemistry is ~0.8 (Björkman and Demmig, 1987). At the Qo site of Cyt *bf*, 2 molecules of PQH_2_ release 4 protons to the lumen and transfer 2 electrons toward Pc. This yield of protons and electrons are doubled by means of Q cycle mechanism (Sacksteder et al., 2000). After all, 12 protons are released to the lumen and 4 Pc and then 4 NADP^+^ are reduced by LEF. Protons released to the lumen are used to rotate the proton turbine in the CFo subcomplex of chloroplast ATP synthase, where the CF_1_ subcomplex phosphorylates 3 ADP molecules per 1 rotation of the CFo. Because the number of proton-binding c-subunit in the CFo is 14 (Seelert et al., 2000), 12 protons generated by LEF can only synthesize 2.6 molecules of ATP. In order to synthesize 3 molecules of ATP, 1 electron from PSI thus needs to be reused by Cyt *bf*, namely CEF, to achieve an ATP: NADPH ratio of (3:2). CEF is thus essential for photosynthetic organisms to run productive electron transport (Munekage et al., 2004). The scheme described above manifests the quantum yield of PSII, where 5 photons are required for 4 turnovers, is lower than that of PSI, where 5 photons are required for 5 turnovers (Figure 4). When light energy is equally distributed between the two photosystems, the PSII quantum yield is 20% lower than PSI because 20% of electrons on PSI are circulating in the CEF pathway. Since ATP and NADPH could be consumed in various cellular reactions, each demand fluctuates from time to time so that photosynthetic organisms need to constantly adjust the relative ratio of the two electron flow modes. Furthermore, CEF has another crucial role, namely building high proton motive force across the thylakoid membrane, which is especially important under adverse environmental conditions to induce NPQ mechanism as described in the previous section. Chloroplasts thus respond to the energy status and stress conditions of the cell by modulating the rate of CEF.

In *C. reinhardtii*, it has been suggested that the modulation of CEF is linked to state transitions (Finazzi et al., 2002). When the light-induced reduction of Cyt *bf* was examined in State 1- and State 2-adapted cells, differential sensitivity to the PSII inhibitor 3-(3,4-dichlorophenyl)-1,1-dimethylurea (DCMU) was observed. DCMU blocked the reduction of Cyt *bf* in State 1, but not in State 2, whereas sensitivity to the inhibitor of Cyt *bf* 2,5-dibromo-3-methyl-6-isopropylbenzoquinone (DBMIB) was identical in State 1 and State 2, suggesting that PSII-independent but CEF-dependent Cyt *bf* reduction occurred only in State 2 (Finazzi et al., 1999). Furthermore, in the *stt7* mutant, which is locked in State 1 because of the lack of kinase for LHCII phosphorylation (Depège et al., 2003), electron flow remained sensitive to DCMU even under conditions in which the PQ pool was reduced (Finazzi et al., 2002). Thus, it seems likely that upon preferential excitation of PSII (State 2), CEF becomes predominant, whereas LEF predominates upon preferential excitation of PSI (State 1).

Recently, the machinery for CEF was biochemically identified by utilizing the conditions leading to State 2 (Iwai et al., 2010). Thylakoid membranes from WT *C. reinhardtii* cells were first treated with carbonyl cyanide 4-(trifluoromethoxy)phenylhydrazone (FCCP), which depletes intracellular ATP pools by impairing respiration and stimulates glycolysis according to the Pasteur effect (Bulté et al., 1990), causing the stroma, and then the PQ pool, to be reduced. Among the solubilized membrane supercomplexes, a super-supercomplex composed of the PSI-LHCI supercomplex with LHCIIs, Cyt *bf*, Fd-NADPH oxidoreductase (FNR), and an integral membrane protein PGR5-like 1(PGRL1) (Dalcorso et al., 2008) was found in a fraction heavier than the PSI-LHCI supercomplex (Iwai et al., 2010). Spectroscopic analyses indicated that upon illumination, reducing equivalents downstream of PSI are transferred to Cyt *bf*, while the oxidized PSI is re-reduced by reducing equivalents from Cyt *bf*, suggesting that this supercomplex is engaged in CEF (Iwai et al., 2010) (Figure 3). The redox carriers (e.g., PQ, Cyt bf, Pc, PSI, Fd, and FNR) shared in both CEF and LEF are potentially in competition with one another. Furthermore, the redox poise of the CEF components could be disturbed if reduced LEF components coexist. Therefore, formation of a PSI-LHCI-LHCII-FNR-Cyt *bf*-PGRL1 super-supercomplex would be advantageous because it could compartmentalize CEF by localizing the mobile electron carriers (PQ, Fd, and Pc), which would allow for the production of a functional pool of CEF components to sustain the increased CEF activity.

Further reports have provided more information about CEF in *C. reinhardtii*. Mutants in which three thylakoid membrane proteins including PgrL1, CAS (Ca^2+^-sensor protein), and ANR1 (anaerobic response protein 1) were independently knocked down were found to have decreased CEF activity under anaerobic conditions (Terashima et al., 2012). A knock-out mutant of PgrL1 was also shown to have decreased CEF activity (Tolleter et al., 2011). Because these three proteins were detected in the CEF supercomplex, they are likely to be additional components of the CEF supercomplex under anaerobic conditions (Terashima et al., 2012). The capacity for state transitions, however, is unaffected in these mutants, so state transitions are probably not causally related to LEF/CEF switching, but rather coincidental (Terashima et al., 2012). This was supported by examining CEF activity in the State 2-locked mutant of *C. reinhardtii* (Takahashi et al., 2013). Although lateral migration of mobile LHCIIs occurred in the *ptox2* mutant, which was State 2-locked because of a lack of plastid terminal oxidase 2 (Houille-Vernes et al., 2011), CEF was negligible, much like the WT in State 1. Furthermore, WT and *ptox2*, as well as the State 1-locked *stt7*, contained CEF supercomplexes under anaerobic conditions, but not under aerobic conditions, indicating that a reduced environment in the stroma, which was experimentally generated under anaerobic conditions, is more likely than LHCII phosphorylation to be the trigger for the LEF/CEF switch. Current understanding of LEF/CEF switching can be summarized as follows: Upon reduction of the stroma, CEF is activated and causes the PQ pool to be more reduced, which then induces a State 1-to-2 transition if Stt7 kinase is present (Figure 2). As discussed by Takahashi et al. (2013), the mechanisms by which reducing power promotes CEF and formation of the CEF supercomplexes remain to be unraveled at the molecular level.

Last but not least to discuss is the intriguing CEF component PgrL1. PgrL1 was first described as a thylakoid integral membrane protein, a mutant deficient in which displayed a similar phenotype as *pgr5* of *A. thaliana* (Dalcorso et al., 2008), which was originally reported as an allele affecting CEF in *A. thaliana*. From among NPQ mutant lines, Munekage et al. (2002) isolated a mutant that has restricted reduction rates for P700^+^ and PQ pool under HL conditions. Since CEF was characterized in early studies as antimycin A-sensitive (Tagawa et al., 1963), an elusive antimycin A-binding enzyme Fd-PQ oxidoreductase (FQR) was proposed to bypasses Cyt *bf* and interface with PQ pool reduction by Fd (Bendall and Manasse, 1995). Because *pgr5* affected the antimycin A-sensitive electron transfer pathway, Pgr5 was assumed to be a part of FQR (Shikanai, 2007). Upon oxidation of its redox-active cysteine residues, PgrL1 from *A. thaliana* forms a complex with PGR5. This PgrL1-Pgr5 complex is proposed to associate with PSI and is reduced by Fd in a PGR5-dependent manner; the reduced PGRL1 may then be monomerized and migrate toward Cyt *bf* where it reduces PQ in an antimycin A-sensitive fashion (Hertle et al., 2013). Whereas the genes for both PGR5 and PGRL1 are present in the genome of *C. reinhardtii* (Merchant et al., 2007), only PGRL1 is included in the CEF supercomplex (Iwai et al., 2010). Protein expression of PGR5 in *C. reinhardtii* has not been confirmed under the conditions so far tested. This difference between a vascular plant and a green alga might reflect a difference in the primary mechanism for CEF between the two organisms.

## Concluding remarks

The availability of a phenomenal crystal structure of the PSII core complex at 1.9 Å (Umena et al., 2011) demonstrates that our journey of photosynthesis research in the static world is approaching its goal, which in turn opens the door to the world of dynamism. Now that the static world is becoming crystal clear, exploring the dynamic world represents one of the next challenges for biologists. Photosynthesis is dynamically regulated in nature, as required by environmental and developmental cues. However, such phenomena are extremely complex and their exploration has inevitably been difficult. Thanks to recent technical advances, including a large variety of mutant banks, purification methodology for large membrane supercomplexes, powerful proteomic approaches aided by genomic information, and computer-aided electron microscopy, those complex research topics are now within our reach.

The idea of acclimation via supramolecular reorganization of protein complexes is not particularly new, nor is it unique in the field of biology. However, what takes place in thylakoids is a large-scale and dynamic reorganization of the supercomplexes and because those are in most cases triggered simply by special light cues, the events in their entirety are truly amazing. We see a typical example in the recent study of HL-grown *C. reinhardtii* cells (Allorent et al., 2013). The respective roles of qE and qT in photoprotection were studied in mutants (*npq4*, *stt7*, *npq4*/*stt7*) and WT. Both state transitions and qE were induced by HL and the double mutant exhibited increased photosensitivity with respect to the single mutants and the WT, suggesting that besides qE, state transitions also play a photoprotective role during HL acclimation. In addition, a line of evidence for “state transition-dependent migration of the qE effector LHCSR3” was presented (Allorent et al., 2013).

In this article, I have summarized the various approaches that have been utilized during the last 10 years or so. State transitions, excess energy dissipation, and CEF have been paid particular attention by researchers and numerous new findings are being reported. The green alga *C. reinhardtii* and the vascular plant *A. thaliana* are the two almost exclusively studied organisms in this new area. The accumulated findings indicate there are several differences in the results from these two species, for instance in the role of PgrL1 in CEF, the identity of the qE effector, and the significance of state transitions. Whether the differences can be explained only in evolutionary terms or they are instead merely superficial differences and represent small parts within some unified mechanisms are expected to be clarified in the near term.

### Conflict of interest statement

The author declares that the research was conducted in the absence of any commercial or financial relationships that could be construed as a potential conflict of interest.

## Abbreviations

Chl: chlorophyll
CEF: cyclic electron flow
α-DM: n-dodecyl-α-D-maltoside
β-DM: n-dodecyl-β-D-maltoside
DBMIB: 2,5-dibromo-3-methyl-6-isopropylbenzoquinone
Cyt *bf*: cytochrome *b*_6_*f* complex
DCMU: 3-(3,4-Dichlorophenyl)-1,1-dimethylurea
FCCP: carbonyl cyanide 4-(trifluoromethoxy)phenylhydrazone
Fd: ferredoxin
FNR: ferredoxin-NADPH oxidoreductase
HL: high light
LHCI and LHCII: light-harvesting complex protein I and II
LEF: linear electron flow
LL: low light
NPQ: non-photochemical quenching
Pc: plastocyanin
PSI and PSII: photosystem I and II
Q: plastoquinone
WT: wild type.

## References

[B1] Ahn T. K. Avenson T. J. Ballottari M. Cheng Y.-C. Niyogi K. K. Bassi R. (2008). Architecture of a charge-transfer state regulating light harvesting in a plant antenna protein. Science 320, 794–797 10.1126/science.1154800 18467588

[B2] Allen J. F. (1992). Protein phosphorylation in regulation of photosynthesis. Biochim. Biophys. Acta 1098, 275–335 10.1016/S0005-2728(09)91014-3 1310622

[B3] Allen J. F. (2003). Cyclic, pseudocyclic and noncyclic photophosphorylation: new links in the chain. Trends Plant Sci. 8, 15–19 10.1016/S1360-1385(02)00006-7 12523995

[B4] Allorent G. Tokutsu R. Roach T. Peers G. Cardol P. Girard-Bascou J. (2013). A dual strategy to cope with high light in *Chlamydomonas reinhardtii*. Plant Cell 25, 545–557 10.1105/tpc.112.108274 23424243 PMC3608777

[B5] Alric J. (2010). Cyclic electron flow around photosystem I in unicellular green algae. Photosynth. Res. 106, 47–56 10.1007/s11120-010-9566-4 20532629

[B6] Amunts A. Toporik H. Borovikova A. Nelson N. (2010). Structure determination and improved model of plant photosystem I. J. Biol. Chem. 285, 3478–3486 10.1074/jbc.M109.072645 19923216 PMC2823434

[B7] Anwaruzzaman M. Chin B. L. Li X. P. Lohr M. Martinez D. A. Niyogi K. K. (2004). Genomic analysis of mutants affecting xanthophyll biosynthesis and regulation of photosynthetic light harvesting in *Chlamydomonas reinhardtii*. Photosynth. Res. 82, 265–276 10.1007/s11120-004-2439-y 16143839

[B8] Arnon D. I. Whatley F. R. Allen M. B. (1958). Assimilatory power in photosynthesis: photosynthetic phosphorylation by isolated chloroplasts is coupled with TPN reduction. Science 127, 1026–1034 10.1126/science.127.3305.1026 17793272

[B9] Bassi R. Rigoni F. Barbato R. Giacometti G. M. (1988). Light-harvesting chlorophyll *a*/*b* proteins (LHCII) populations in phosphorylated membranes. Biochim. Biophys. Acta 936, 29–38 10.1016/0005-2728(88)90248-4

[B10] Bellafiore S. Barneche F. Peltier G. Rochaix J.-D. (2005). State transitions and light adaptation require chloroplast thylakoid protein kinase STN7. Nature 433, 892–895 10.1038/nature03286 15729347

[B11] Bendall D. S. Manasse R. S. (1995). Cyclic photophosphorylation and electron transport. Biochim. Biophys. Acta 1229, 23–38 10.1016/0005-2728(94)00195-B

[B12] Betterle N. Ballottari M. Zorzan S. de Bianchi S. Cazzaniga S. Dall'osto L. (2009). Light-induced dissociation of an antenna hetero-oligomer is needed for non-photochemical quenching induction. J. Biol. Chem. 284, 15255–15266 10.1074/jbc.M808625200 19307183 PMC2685706

[B13] Björkman O. Demmig B. (1987). Photon yield of O_2_ evolution and chlorophyll fluorescence characteristics at 77 K among vascular plants of diverse origins. Planta 170, 489–504 10.1007/BF00402983 24233012

[B14] Boekema E. J. Dekker J. P. van Heel M. G. Rögner M. Saenger W. Witt I. (1987). Evidence for a trimeric organization of the photosystem I complex from the thermophilic cyanobacterium *Synechococcus* sp. FEBS Lett. 217, 283–286 10.1016/0014-5793(87)80679-8

[B15] Boekema E. J. Hankamer B. Bald D. Kruip J. Nield J. Boonstra A. F. (1995). Supramolecular structure of the photosystem II complex from green plants and cyanobacteria. Proc. Natl. Acad. Sci. U.S.A. 92, 175–179 10.1073/pnas.92.1.175 7816811 PMC42840

[B16] Boekema E. J. Jensen P. E. Schlodder E. van Breemen J. F. Van Roon H. Scheller H. V. (2001). Green plant photosystem I binds light-harvesting complex I on one side of the complex. Biochemistry 40, 1029–1036 10.1021/bi0015358 11170425

[B17] Boekema E. J. Nield J. Hankamer B. Barber J. (1998). Localization of the 23-kDa subunit of the oxygen-evolving complex of photosystem II by electron microscopy. Eur. J. Biochem. 252, 268–276 10.1046/j.1432-1327.1998.2520268.x 9523698

[B18] Boekema E. J. Van Roon H. Van Breemen J. F. Dekker J. P. (1999). Supramolecular organization of photosystem II and its light-harvesting antenna in partially solubilized photosystem II membranes. Eur. J. Biochem. 266, 444–452 10.1046/j.1432-1327.1999.00876.x 10561584

[B19] Bonardi V. Pesaresi P. Becker T. Schleiff E. Wagner R. Pfannschmidt T. (2005). Photosystem II core phosphorylation and photosynthetic acclimation require two different protein kinases. Nature 437, 1179–1182 10.1038/nature04016 16237446

[B20] Bonaventura C. Myers J. (1969). Fluorescence and oxygen evolution from *Chlorella pyrenoidosa*. Biochim. Biophys. Acta 189, 366–383 10.1016/0005-2728(69)90168-6 5370012

[B21] Bonente G. Ballottari M. Truong T. B. Morosinotto T. Ahn T. K. Fleming G. R. (2011). Analysis of LhcSR3, a protein essential for feedback de-excitation in the green alga *Chlamydomonas reinhardtii*. PLoS Biol. 9:e1000577 10.1371/journal.pbio.1000577 21267060 PMC3022525

[B22] Bonente G. Howes B. D. Caffarri S. Smulevich G. Bassi R. (2008). Interactions between the photosystem II subunit PsbS and xanthophylls studied *in vivo* and *in vitro*. J. Biol. Chem. 283, 8434–8445 10.1074/jbc.M708291200 18070876 PMC2417184

[B23] Bulté L. Gans P. Rebéillé F. Wollman F.-A. (1990). ATP control on state transitions *in vivo* in *Chlamydomonas reinhardtii*. Biochim. Biophys. Acta 1020, 72–80 10.1016/0005-2728(90)90095-L

[B24] Caffarri S. Kouril R. Kereiche S. Boekema E. J. Croce R. (2009). Functional architecture of higher plant photosystem II supercomplexes. EMBO J. 28, 3052–3063 10.1038/emboj.2009.232 19696744 PMC2760109

[B25] Dalcorso G. Pesaresi P. Masiero S. Aseeva E. Schunemann D. Finazzi G. (2008). A complex containing PGRL1 and PGR5 is involved in the switch between linear and cyclic electron flow in *Arabidopsis*. Cell 132, 273–285 10.1016/j.cell.2007.12.028 18243102

[B26] Damkjaer J. T. Kereiche S. Johnson M. P. Kovacs L. Kiss A. Z. Boekema E. J. (2009). The photosystem II light-harvesting protein Lhcb3 affects the macrostructure of photosystem II and the rate of state transitions in Arabidopsis. Plant Cell 21, 3245–3256 10.1105/tpc.108.064006 19880802 PMC2782274

[B27] Daum B. Nicastro D. Austin J. 2nd. McIntosh J. R. Kühlbrandt W. (2010). Arrangement of photosystem II and ATP synthase in chloroplast membranes of spinach and pea. Plant Cell 22, 1299–1312 10.1105/tpc.109.071431 20388855 PMC2879734

[B28] de Bianchi S. Ballottari M. Dall'osto L. Bassi R. (2010). Regulation of plant light harvesting by thermal dissipation of excess energy. Biochem. Soc. Trans. 38, 651–660 10.1042/BST0380651 20298238

[B29] de Bianchi S. Dall'osto L. Tognon G. Morosinotto T. Bassi R. (2008). Minor antenna proteins CP24 and CP26 affect the interactions between photosystem II subunits and the electron transport rate in grana membranes of *Arabidopsis*. Plant Cell 20, 1012–1028 10.1105/tpc.107.055749 18381925 PMC2390724

[B30] Dekker J. P. Boekema E. J. (2005). Supramolecular organization of thylakoid membrane proteins in green plants. Biochim. Biophys. Acta 1706, 12–39 10.1016/j.bbabio.2004.09.009 15620363

[B31] Delosme R. Olive J. Wollman F.-A. (1996). Changes in light energy distribution upon state transitions: an *in vivo* photoacoustic study of the wild type and photosynthesis mutants from *Chlamydomonas reinhardtii*. Biochim. Biophys. Acta 1273, 150–158 10.1016/0005-2728(95)00143-3

[B32] Demmig-Adams B. Adams W.W. III (1992). Photoprotection and other responses of plants to high light stress. Annu. Rev. Plant Biol. Plant Mol. Biol. 43, 599–626 10.1146/annurev.pp.43.060192.003123

[B33] Demmig-Adams B. Ebbert V. Mellman D. L. Mueh K. E. Schaffer L. Funk C. (2006). Modulation of PsbS and flexible vs sustained energy dissipation by light environment in different species. Physiol. Plant 127, 670–680 10.1111/j.1399-3054.2006.00698.x

[B34] Depège N. Bellafiore S. Rochaix J.-D. (2003). Role of chloroplast protein kinase Stt7 in LHCII phosphorylation and state transition in *Chlamydomonas*. Science 299, 1572–1575 10.1126/science.1081397 12624266

[B35] Dietzel L. Brautigam K. Steiner S. Schuffler K. Lepetit B. Grimm B. (2011). Photosystem II supercomplex remodeling serves as an entry mechanism for state transitions in *Arabidopsis*. Plant Cell 23, 2964–2977 10.1105/tpc.111.087049 21880991 PMC3180804

[B36] Drop B. Webber-Birungi M. Fusetti F. Kouril R. Redding K. E. Boekema E. J. (2011). Photosystem I of *Chlamydomonas reinhardtii* is composed of nine light-harvesting complexes (Lhca) located on one side of the core. J. Biol. Chem. 286, 44878–44887 10.1074/jbc.M111.301101 22049081 PMC3247965

[B37] Elrad D. Niyogi K. K. Grossman A. R. (2002). A major light-harvesting polypeptide of photosystem II functions in thermal dissipation. Plant Cell 14, 1801–1816 10.1105/tpc.002154 12172023 PMC151466

[B38] Finazzi G. (2005). The central role of the green alga *Chlamydomonas reinhardtii* in revealing the mechanism of state transitions. J. Exp. Bot. 56, 383–388 10.1093/jxb/erh230 15333639

[B39] Finazzi G. Furia A. Barbagallo R. P. Forti G. (1999). State transitions, cyclic and linear electron transport and photophosphorylation in *Chlamydomonas reinhardtii*. Biochim. Biophys. Acta 1413, 117–129 10.1016/S0005-2728(99)00089-4 10556624

[B40] Finazzi G. Johnson G. N. Dall'osto L. Zito F. Bonente G. Bassi R. (2006). Nonphotochemical quenching of chlorophyll fluorescence in *Chlamydomonas reinhardtii*. Biochemistry 45, 1490–1498 10.1021/bi0521588 16445291

[B41] Finazzi G. Rappaport F. Furia A. Fleischmann M. Rochaix J.-D. Zito F. (2002). Involvement of state transitions in the switch between linear and cyclic electron flow in *Chlamydomonas reinhardtii*. EMBO Rep. 3, 280–285 10.1093/embo-reports/kvf047 11850400 PMC1084013

[B42] Förster B. Osmond C. B. Boynton J. E. (2001). Very high light resistant mutants of *Chlamydomonas reinhardtii*: responses of photosystem II, nonphotochemical quenching and xanthophyll pigments to light and CO_2_. Photosynth. Res. 67, 5–15 10.1023/A:1010611509209 16228312

[B43] Gagne G. Guertin M. (1992). The early genetic response to light in the green unicellular alga *Chlamydomonas eugametos* grown under light/dark cycles involves genes that represent direct responses to light and photosynthesis. Plant Mol. Biol. 18, 429–445 10.1007/BF00040659 1371402

[B44] Galka P. Santabarbara S. Khuong T. T. Degand H. Morsomme P. Jennings R. C. (2012). Functional analyses of the plant photosystem I-light-harvesting complex II supercomplex reveal that light-harvesting complex II loosely bound to photosystem II is a very efficient antenna for photosystem I in state II. Plant Cell 24, 2963–2978 10.1105/tpc.112.100339 22822202 PMC3426126

[B45] Germano M. Yakushevska A. E. Keegstra W. van Gorkom H. J. Dekker J. P. Boekema E. J. (2002). Supramolecular organization of photosystem I and light-harvesting complex I in *Chlamydomonas reinhardtii*. FEBS Lett. 525, 121–125 10.1016/S0014-5793(02)03100-9 12163173

[B46] Goral T. K. Johnson M. P. Duffy C. D. Brain A. P. Ruban A. V. Mullineaux C. W. (2012). Light-harvesting antenna composition controls the macrostructure and dynamics of thylakoid membranes in Arabidopsis. Plant J. 69, 289–301 10.1111/j.1365-313X.2011.04790.x 21919982

[B47] Gutman B. L. Niyogi K. K. (2004). *Chlamydomonas* and *Arabidopsis*. A dynamic duo. Plant Physiol. 135, 607–610 10.1104/pp.104.041491 15208408 PMC514095

[B48] Hankamer B. Nield J. Zheleva D. Boekema E. Jansson S. Barber J. (1997). Isolation and biochemical characterisation of monomeric and dimeric photosystem II complexes from spinach and their relevance to the organisation of photosystem II *in vivo*. Eur. J. Biochem. 243, 422–429 10.1111/j.1432-1033.1997.0422a.x 9030768

[B49] Harrer R. Bassi R. Testi M. G. Schäfer C. (1998). Nearest-neighbor analysis of a photosystem II complex from *Marchantia polymorpha* L. (liverwort), which contains reaction center and antenna proteins. Eur. J. Biochem. 255, 196–205 10.1046/j.1432-1327.1998.2550196.x 9692919

[B50] Hertle A. P. Blunder T. Wunder T. Pesaresi P. Pribil M. Armbruster U. (2013). PGRL1 is the elusive ferredoxin-plastoquinone reductase in photosynthetic cyclic electron flow. Mol. Cell 49, 511–523 10.1016/j.molcel.2012.11.030 23290914

[B51] Horton P. Johnson M. P. Perez-Bueno M. L. Kiss A. Z. Ruban A. V. (2008). Photosynthetic acclimation: does the dynamic structure and macro-organisation of photosystem II in higher plant grana membranes regulate light harvesting states? FEBS J. 275, 1069–1079 10.1111/j.1742-4658.2008.06263.x 18318834

[B52] Horton P. Ruban A. V. Walters R. G. (1996). Regulation of light harvesting in green plants. Annu. Rev. Plant Biol. Plant Mol. Biol. 47, 655–684 10.1146/annurev.arplant.47.1.655 15012304

[B53] Houille-Vernes L. Rappaport F. Wollman F.-A. Alric J. Johnson X. (2011). Plastid terminal oxidase 2 (PTOX2) is the major oxidase involved in chlororespiration in *Chlamydomonas*. Proc. Natl. Acad. Sci. U.S.A. 108, 20820–20825 10.1073/pnas.1110518109 22143777 PMC3251066

[B54] Iwai M. Takahashi Y. Minagawa J. (2008). Molecular remodeling of photosystem II during state transitions in *Chlamydomonas reinhardtii*. Plant Cell 20, 2177–2189 10.1105/tpc.108.059352 18757554 PMC2553614

[B55] Iwai M. Takizawa K. Tokutsu R. Okamuro A. Takahashi Y. Minagawa J. (2010). Isolation of the elusive supercomplex that drives cyclic electron flow in photosynthesis. Nature 464, 1210–1213 10.1038/nature08885 20364124

[B56] Jansson S. (1999). A guide to the *Lhc* genes and their relatives in *Arabidopsis*. Trends Plant Sci. 4, 236–240 10.1016/S1360-1385(99)01419-3 10366881

[B57] Jensen P. E. Haldrup A. Zhang S. Scheller H. V. (2004). The PSI-O subunit of plant photosystem I is involved in balancing the excitation pressure between the two photosystems. J. Biol. Chem. 279, 24212–24217 10.1074/jbc.M403147200 15169790

[B58] Johnson G. N. (2011). Physiology of PSI cyclic electron transport in higher plants. Biochim. Biophys. Acta 1807, 384–389 10.1016/j.bbabio.2010.11.009 21118673

[B59] Johnson M. P. Goral T. K. Duffy C. D. Brain A. P. Mullineaux C. W. Ruban A. V. (2011). Photoprotective energy dissipation involves the reorganization of photosystem II light-harvesting complexes in the grana membranes of spinach chloroplasts. Plant Cell 23, 1468–1479 10.1105/tpc.110.081646 21498680 PMC3101555

[B60] Jordan P. Fromme P. Witt H. T. Klukas O. Saenger W. Krauss N. (2001). Three-dimensional structure of cyanobacterial photosystem I at 2.5 Å resolution. Nature 411, 909–917 10.1038/35082000 11418848

[B61] Kargul J. Turkina M. V. Nield J. Benson S. Vener A. V. Barber J. (2005). Light-harvesting complex II protein CP29 binds to photosystem I of *Chlamydomonas reinhardtii* under State 2 conditions. FEBS J. 272, 4797–4806 10.1111/j.1742-4658.2005.04894.x 16156798

[B62] Kereiche S. Kiss A. Z. Kouril R. Boekema E. J. Horton P. (2010). The PsbS protein controls the macro-organisation of photosystem II complexes in the grana membranes of higher plant chloroplasts. FEBS Lett. 584, 759–764 10.1016/j.febslet.2009.12.031 20035752

[B63] Kiss A. Z. Ruban A. V. Horton P. (2008). The PsbS protein controls the organization of the photosystem II antenna in higher plant thylakoid membranes. J. Biol. Chem. 283, 3972–3978 10.1074/jbc.M707410200 18055452

[B64] Kouřil R. Oostergetel G. T. Boekema E. J. (2011). Fine structure of granal thylakoid membrane organization using cryo electron tomography. Biochim. Biophys. Acta 1807, 368–374 10.1016/j.bbabio.2010.11.007 21110939

[B65] Kovács L. Damkjær J. Kereïche S. Ilioaia C. Ruban A. V. Boekema E. J. (2006). Lack of the light-harvesting complex CP24 affects the structure and function of the grana membranes of higher plant chloroplasts. Plant Cell 18, 3106–3120 10.1105/tpc.106.045641 17114352 PMC1693946

[B66] Kramer D. M. Avenson T. J. Edwards G. E. (2004). Dynamic flexibility in the light reactions of photosynthesis governed by both electron and proton transfer reactions. Trends Plant Sci. 9, 349–357 10.1016/j.tplants.2004.05.001 15231280

[B67] Lemeille S. Rochaix J. D. (2010). State transitions at the crossroad of thylakoid signalling pathways. Photosynth. Res. 106, 33–46 10.1007/s11120-010-9538-8 20217232

[B68] Li X. P. Björkman O. Shih C. Grossman A. R. Rosenquist M. Jansson S. (2000). A pigment-binding protein essential for regulation of photosynthetic light harvesting. Nature 403, 391–395 10.1038/35000131 10667783

[B69] Li X. P. Gilmore A. M. Caffarri S. Bassi R. Golan T. Kramer D. (2004). Regulation of photosynthetic light harvesting involves intrathylakoid lumen pH sensing by the PsbS protein. J. Biol. Chem. 279, 22866–22874 10.1074/jbc.M402461200 15033974

[B70] Li Z. Wakao S. Fischer B. B. Niyogi K. K. (2009). Sensing and responding to excess light. Annu. Rev. Plant Biol. 60, 239–260 10.1146/annurev.arplant.58.032806.103844 19575582

[B71] Lunde C. Jensen P. E. Haldrup A. Knoetzel J. Scheller H. V. (2000). The PSI-H subunit of photosystem I is essential for state transitions in plant photosynthesis. Nature 408, 613–615 10.1038/35046121 11117752

[B72] Merchant S. S. Prochnik S. E. Vallon O. Harris E. H. Karpowicz S. J. Witman G. B. (2007). The *Chlamydomonas* genome reveals the evolution of key animal and plant functions. Science 318, 245–250 10.1126/science.1143609 17932292 PMC2875087

[B73] Minagawa J. (2009). Light-harvesting proteins, in Chlamydomonas Sourcebook, eds Stern D. Harris E. H. (Amsterdam: Springer), 503–540 10.1016/B978-0-12-370873-1.00022-8

[B74] Minagawa J. (2011). State transitions–the molecular remodeling of photosynthetic supercomplexes that controls energy flow in the chloroplast. Biochim. Biophys. Acta 1807, 897–905 10.1016/j.bbabio.2010.11.005 21108925

[B75] Minagawa J. Takahashi Y. (2004). Structure, function and assembly of photosystem II and its light-harvesting proteins. Photosynth. Res. 82, 241–263 10.1007/s11120-004-2079-2 16143838

[B76] Miura K. Yamano T. Yoshioka S. Kohinata T. Inoue Y. Taniguchi F. (2004). Expression profiling-based identification of CO_2_-responsive genes regulated by CCM1 controlling a carbon-concentrating mechanism in *Chlamydomonas reinhardtii*. Plant Physiol. 135, 1595–1607 10.1104/pp.104.041400 15235119 PMC519074

[B77] Müller P. Li X. P. Niyogi K. K. (2001). Non-photochemical quenching. A response to excess light energy. Plant Physiol. 125, 1558–1566 10.1104/pp.125.4.1558 11299337 PMC1539381

[B78] Munekage Y. Hashimoto M. Miyake C. Tomizawa K. Endo T. Tasaka M. (2004). Cyclic electron flow around photosystem I is essential for photosynthesis. Nature 429, 579–582 10.1038/nature02598 15175756

[B79] Munekage Y. Hojo M. Meurer J. Endo T. Tasaka M. Shikanai T. (2002). PGR5 is involved in cyclic electron flow around photosystem I and is essential for photoprotection in *Arabidopsis*. Cell 110, 361–371 10.1016/S0092-8674(02)00867-X 12176323

[B80] Murata N. (1969). Control of excitation transfer in photosynthesis. I. Light-induced change of chlorophyll a fluoresence in *Porphyridium cruentum*. Biochim. Biophys. Acta 172, 242–251 10.1016/0005-2728(69)90067-X 5775694

[B81] Naumann B. Busch A. Allmer J. Ostendorf E. Zeller M. Kirchhoff H. (2007). Comparative quantitative proteomics to investigate the remodeling of bioenergetic pathways under iron deficiency in *Chlamydomonas reinhardtii*. Proteomics 7, 3964–3979 10.1002/pmic.200700407 17922516

[B82] Nield J. Kruse O. Ruprecht J. da Fonseca P. Büchel C. Barber J. (2000a). Three-dimensional structure of *Chlamydomonas reinhardtii* and *Synechococcus elongatus* photosystem II complexes allows for comparison of their oxygen-evolving complex organization. J. Biol. Chem. 275, 27940–27946 10.1074/jbc.M003069200 10807922

[B83] Nield J. Orlova E. V. Morris E. P. Gowen B. van Heel M. Barber J. (2000b). 3D map of the plant photosystem II supercomplex obtained by cryoelectron microscopy and single particle analysis. Nat. Struct. Biol. 7, 44–47 10.1038/71242 10625426

[B84] Niyogi K. K. Björkman O. Grossman A. R. (1997a). *Chlamydomonas* xanthophyll cycle mutants identified by video imaging of chlorophyll fluorescence quenching. Plant Cell 9, 1369–1380 10.1105/tpc.9.8.1369 12237386 PMC157004

[B85] Niyogi K. K. Björkman O. Grossman A. R. (1997b). The roles of specific xanthophylls in photoprotection. Proc. Natl. Acad. Sci. U.S.A. 94, 14162–14167 10.1073/pnas.94.25.14162 9391170 PMC28450

[B85a] Niyogi K. K. Grossman A. R. Björkman O. (1998). *Arabidopsis* mutants define a central role for the xanthophyll cycle in the regulation of photosynthetic energy conversion. Plant Cell 10, 1121–1134 9668132 10.1105/tpc.10.7.1121 PMC144052

[B86] Peers G. Truong T. B. Ostendorf E. Busch A. Elrad D. Grossman A. R. (2009). An ancient light-harvesting protein is critical for the regulation of algal photosynthesis. Nature 462, 518–521 10.1038/nature08587 19940928

[B87] Pesaresi P. Lunde C. Jahns P. Tarantino D. Meurer J. Varotto C. (2002). A stable LHCII-PSI aggregate and suppression of photosynthetic state transitions in the *psae1-1* mutant of *Arabidopsis thaliana*. Planta 215, 940–948 10.1007/s00425-002-0835-0 12355154

[B88] Richard C. Ouellet H. Guertin M. (2000). Characterization of the LI818 polypeptide from the green unicellular alga *Chlamydomonas reinhardtii*. Plant Mol. Biol. 42, 303–316 10.1023/A:1006340308077 10794530

[B89] Ruban A. V. Berera R. Ilioaia C. van Stokkum I. H. Kennis J. T. Pascal A. A. (2007). Identification of a mechanism of photoprotective energy dissipation in higher plants. Nature 450, 575–578 10.1038/nature06262 18033302

[B90] Ruban A. V. Wentworth M. Yakushevska A. E. Andersson J. Lee P. J. Keegstra W. (2003). Plants lacking the main light-harvesting complex retain photosystem II macro-organization. Nature 421, 648–652 10.1038/nature01344 12571599

[B91] Sacksteder C. A. Kanazawa A. Jacoby M. E. Kramer D. M. (2000). The proton to electron stoichiometry of steady-state photosynthesis in living plants: a proton-pumping Q cycle is continuously engaged. Proc. Natl. Acad. Sci. U.S.A. 97, 14283–14288 10.1073/pnas.97.26.14283 11121034 PMC18910

[B92] Seelert H. Poetsch A. Dencher N. A. Engel A. Stahlberg H. Muller D. J. (2000). Structural biology. Proton-powered turbine of a plant motor. Nature 405, 418–419 10.1038/35013148 10839529

[B93] Shikanai T. (2007). Cyclic electron transport around photosystem I: genetic approaches. Annu. Rev. Plant Biol. 58, 199–217 10.1146/annurev.arplant.58.091406.110525 17201689

[B94] Tagawa K. Tsujimoto H. Y. Arnon D. I. (1963). Role of chloroplast ferredoxin in the energy conversion process of photosynthesis. Proc. Natl. Acad. Sci. U.S.A. 49, 567–572 10.1073/pnas.49.4.567 13980171 PMC299906

[B95] Takahashi H. Clowez S. Wollman F. A. Vallon O. Rappaport F. (2013). Cyclic electron flow is redox-controlled but independent of state transition. Nat. Commun. 4, 1954 10.1038/ncomms2954 23760547 PMC3709502

[B96] Takahashi H. Iwai M. Takahashi Y. Minagawa J. (2006). Identification of the mobile light-harvesting complex II polypeptides for state transitions in *Chlamydomonas reinhardtii*. Proc. Natl. Acad. Sci. U.S.A. 103, 477–482 10.1073/pnas.0509952103 16407170 PMC1326185

[B97] Teramoto H. Ono T.-A. Minagawa J. (2001). Identification of *Lhcb* gene family encoding the light-harvesting chlorophyll-*a*/*b* proteins of photosystem II in *Chlamydomonas reinhardtii*. Plant Cell Physiol. 42, 849–856 10.1093/pcp/pce115 11522911

[B98] Terashima M. Petroutsos D. Hudig M. Tolstygina I. Trompelt K. Gabelein P. (2012). Calcium-dependent regulation of cyclic photosynthetic electron transfer by a CAS, ANR1, and PGRL1 complex. Proc. Natl. Acad. Sci. U.S.A. 109, 17717–17722 10.1073/pnas.1207118109 23045639 PMC3491457

[B99] Tokutsu R. Iwai M. Minagawa J. (2009). CP29, a monomeric light-harvesting complex II protein, is essential for state transitions in *Chlamydomonas reinhardtii*. J. Biol. Chem. 284, 7777–7782 10.1074/jbc.M809360200 19144643 PMC2658071

[B100] Tokutsu R. Kato N. Bui K. H. Ishikawa T. Minagawa J. (2012). Revisiting the supramolecular organization of photosystem II in *Chlamydomonas reinhardtii*. J. Biol. Chem. 287, 31574–31581 10.1074/jbc.M111.331991 22801422 PMC3438989

[B101] Tokutsu R. Minagawa J. (2013). Energy-dissipative supercomplex of photosystem II associated with LHCSR3 in *Chlamydomonas reinhardtii*. Proc. Natl. Acad. Sci. U.S.A. 110, 10016–10021 10.1073/pnas.1222606110 23716695 PMC3683755

[B102] Tolleter D. Ghysels B. Alric J. Petroutsos D. Tolstygina I. Krawietz D. (2011). Control of hydrogen photoproduction by the proton gradient generated by cyclic electron flow in *Chlamydomonas reinhardtii*. Plant Cell 23, 2619–2630 10.1105/tpc.111.086876 21764992 PMC3226202

[B103] Turkina M. V. Kargul J. Blanco-Rivero A. Villarejo A. Barber J. Vener A. V. (2006). Environmentally modulated phosphoproteome of photosynthetic membranes in the green alga *Chlamydomonas reinhardtii*. Mol. Cell. Proteomics 5, 1412–1425 10.1074/mcp.M600066-MCP200 16670252

[B104] Umena Y. Kawakami K. Shen J. R. Kamiya N. (2011). Crystal structure of oxygen-evolving photosystem II at a resolution of 1.9 A. Nature 473, 55–60 10.1038/nature09913 21499260

[B105] Vener A. V. van Kan P. J. Rich P. R. Ohad I. I. Andersson B. (1997). Plastoquinol at the quinol oxidation site of reduced cytochrome *bf* mediates signal transduction between light and protein phosphorylation: thylakoid protein kinase deactivation by a single-turnover flash. Proc. Natl. Acad. Sci. U.S.A. 94, 1585–1590 10.1073/pnas.94.4.1585 11038603 PMC19835

[B106] Watanabe M. Kubota H. Wada H. Narikawa R. Ikeuchi M. (2011). Novel supercomplex organization of photosystem I in *Anabaena* and *Cyanophora paradoxa*. Plant Cell Physiol. 52, 162–168 10.1093/pcp/pcq183 21118826

[B107] Wollman F.-A. Lemaire C. (1988). Studies on kinase-controlled state transitions in photosystem II and *b*_6_*f* mutants from *Chlamydomonas reinhardtii* which lack quinone-binding proteins. Biochim. Biophys. Acta 933, 85–94 10.1016/0005-2728(88)90058-8

[B108] Yakushevska A. E. Keegstra W. Boekema E. J. Dekker J. P. Andersson J. Jansson S. (2003). The structure of photosystem II in *Arabidopsis*: localization of the CP26 and CP29 antenna complexes. Biochemistry 42, 608–613 10.1021/bi027109z 12534272

[B109] Zhang S. Scheller H. V. (2004). Light-harvesting complex II binds to several small subunits of photosystem I. J. Biol. Chem. 279, 3180–3187 10.1074/jbc.M311640200 14617624

[B110] Zito F. Finazzi G. Delosme R. Nitschke W. Picot D. Wollman F.-A. (1999). The Qo site of cytochrome *b*_6_*f* complexes controls the activation of the LHCII kinase. EMBO J. 18, 2961–2969 10.1093/emboj/18.11.2961 10357809 PMC1171378

